# Increasing trypsin inhibitor concentrations in growing pig diets compromises growth performance and protein digestibility

**DOI:** 10.1093/jas/skaf256

**Published:** 2025-08-11

**Authors:** Kayla A Miller, Joel D Spencer, Omarh F Mendoza, Hari B Krishnan, Mitchell J Nisley, Nicholas K Gabler

**Affiliations:** Department of Animal Science, Iowa State University, Ames, IA, USA50011; United Animal Health, Sheridan, IN, USA46069; The Maschhoffs LLC., Carlyle, IL, USA62231; Agricultural Research Service, U.S. Department of Agriculture and Divisions of Plant Sciences and Animal Sciences, University of Missouri, Columbia, MO, USA 65211; Department of Animal Science, Iowa State University, Ames, IA, USA50011; Department of Animal Science, Iowa State University, Ames, IA, USA50011

**Keywords:** antinutritional compounds, growing pigs, protein, soybean, trypsin inhibitor

## Abstract

Soybeans contain trypsin inhibitor proteins that are antinutritional and may result in growth performance inhibition through reduced amino acid bioavailability. The objective of this experiment was to determine the level of active trypsin inhibitor units (**TIU**) per milligram (TIU/mg) of complete feed that leads to reductions in growth performance and nitrogen digestibility. Forty-five grower gilts (39.7 ± 2.1 kg BW) were individually penned and fed 1 of the 5 dietary treatments (*n* = 9 pigs/trt) in a completely randomized design for the duration of this 27-d experiment. Diets were fed in meal form and formulated utilizing raw ground soybeans and soybean meal to achieve increasing levels of TIU/mg of complete feed of 0.89, 1.77, 3.55, 6.99, and 11.54 TIU/mg. Analyzed complete diets contained 0.99, 2.23, 3.07, 6.49, and 9.38 TIU/mg. Diets were balanced to the same grams of standardized ileal digestible (**SID**) lysine and SID lysine per megacalorie of metabolizable energy of 2.85 and neutral detergent fiber (**NDF**) concentration of 7.4% utilizing crystalline amino acids, soybean oil, and soybean hulls, respectively. Pig body weight (**BW**) and individual feed intake were collected on days 0, 21, and 27 to calculate average daily gain (**ADG**), average daily feed intake (**ADFI**), and feed efficiency (**G:F**). On day 21 of the experiment, pigs were placed into metabolism stalls for 6 d to determine nitrogen balance and apparent total tract digestibility (**ATTD**) of nitrogen. All data were analyzed with pig as the experimental unit and TIU/mg level as a fixed effect by ANOVA, including linear and quadratic effect contrasts. Average daily gain decreased as dietary concentrations TIU/mg increased (1.07, 1.03, 1.02, 0.91, and 0.83 kg/d, respectively, linear *P* < 0.001). No differences in ADFI (*P* > 0.10) were detected, but G:F decreased (0.48, 0.45, 0.43, 0.41, 0.36, respectively, linear *P* < 0.001) was observed as TIU/mg increased from 0.99 to 9.38 TIU/mg. Final BW of pigs fed 9.38 TIU/mg was reduced by 9.32% compared with 0.99 TIU/mg (62.3 vs. 68.7 kg, linear *P* < 0.001). Nitrogen ATTD decreased when pigs were fed 9.38 TIU/mg compared to 0.99 and 2.23 TIU/mg fed pigs (83.26% vs. 90.29% and 89.57%, respectively, *P* < 0.001). In conclusion, as TIU from soybeans increased in grower pig diets, linear reductions in protein digestibility, growth rates, and feed efficiency were observed.

## Teaser

Soybeans can contain antinutritional factors such as trypsin inhibitors that can restrict protein digestibility and amino acid utilization for lean growth. Growing pigs consuming concentrations above 3 trypsin inhibitor units (TIU) per mg complete feed may have compromised growth, feed efficiency, and protein retention.

## Introduction

Soybeans and soybean coproducts, namely soybean meal, are highly desired plant protein sources in swine diets for their amino acid concentrations and digestibility. However, soybeans contain several antinutritional factors that can impede the digestion, absorption, and bioavailability of nutrients and energy, thereby affecting animal health and performance (Liener, 1976, 1994). It has been recognized that soybean-based products must be heat processed to deactivate and denature certain antinutritional compounds in soybeans (Liener, 1962; Vagadia et al., 2017) prior to feeding to monogastric species. Protease inhibitors are among the most common antinutritional factors in soybeans. However, other naturally occurring compounds, such as lectins, tannins, saponins, β-conglycinin, and glycinin, all may have antinutritional effects and contribute to growth inhibition if not properly deactivated (Liener, 1962, 1994; Krishnan et al., 2009; Cabrera-Orozco et al., 2013; Wang et al., 2014; Yang et al., 2022).

Two protease inhibitors have been identified to have detrimental effects on growth performance potential: Kunitz trypsin inhibitor (**KTi**; Kunitz, 1946) and Bowman-Birk Inhibitor (Bowman, 1944; Birk, 1961). Collectively, KTi and Bowman-Birk Inhibitor (**BBI**) are referred to as trypsin inhibitors. Ultimately, growth performance loss can be attributed to impeding protein digestion (Liener, 1976), pancreatic enlargement in some species (Hasdai and Liener, 1983; Grant et al., 1995; Clarke and Wiseman, 2005), and hypersecretion of enzymes (Schneeman et al., 1977; Liener, 1994; Guillamon et al., 2008; Cabrera-Orozco et al., 2013). Trypsin inhibitors cause a loss of weight in chicks (Palacios et al., 2004), rats (Liener, 1953), and pigs (Yen et al., 1974; Cook et al., 1988).

Previous research has identified growth impairment in pigs when fed diets containing high levels of trypsin inhibitor protein. Many studies have compared soybean variants known to have high and low levels of trypsin inhibitor protein (Yen et al., 1974; Cook et al., 1988; Palacios et al., 2004) or different processing methods aimed to deactivate trypsin inhibitor (Jimenez et al., 1963; Combs et al., 1967), both of which have shown weight reductions of the pigs utilized. To the best of our knowledge, there has been limited investigation involving trypsin inhibitor unit (**TIU**), expressed as TIU per milligram of feed (TIU/mg), content in growing pigs using a titration model. The authors suggested a maximum inclusion level of 2 TIU/mg of complete feed for grow-finish pigs based on various parameters, including growth performance, carcass performance, and fecal consistency (Royer et al., 2022). However, few published studies have validated this inclusion rate and its impact on growth and digestibility, thus leaving a remaining gap in the literature to determine the inclusion level of trypsin inhibitors that negatively affect pig growth performance and nitrogen utilization. Therefore, the objective of this experiment was to determine the concentration at which TIU from soybeans in dietary formulations would impact grower pig growth performance, digestibility, and nitrogen retention. It was hypothesized that dietary concentrations above 3 TIU/mg would attenuate pig growth performance and feed efficiency by reducing protein digestibility and retention.

## Materials and Methods

Research was conducted at the Iowa State University Swine Nutrition Research Farms (Ames, IA) from July to August of 2023. All procedures were approved by the Iowa State University Institutional Animal Care and Use Committee (IACUC protocol #23-111) and adhere to the ethical and humane use of animals for research.

### Dietary treatments and ingredient trypsin inhibitor analysis

Soybean meal, sourced from Indiana, and whole raw soybeans, sourced from central Iowa, were utilized in this experiment. Whole raw soybeans were ground using a hammer mill upon arrival at the farm. Raw ground soybeans and soybean meal samples were sent to a commercial lab (Midwest Laboratories, Omaha, NE) for complete ingredient analysis prior to dietary formulation (Table 1). Five dietary treatments were formulated with increasing levels of TIU ranging from 0.89 to 11.54 TIU/mg complete feed (0.89, 1.77, 3.55, 6.99, and 11.54 TIU/mg). Diets were formulated in accordance with the NRC (2012) nutrient recommendations for the size of gilt utilized and were balanced for the major limiting amino acids, standardized ileal digestible (**SID**) lysine of 0.98%, neutral detergent fiber (**NDF**) of 7.40%, and metabolizable energy of 3,435 kcal/kg utilizing crystalline amino acids, soybean hulls, and soybean oil, respectively. One dietary phase was fed for the entire duration of the 27-d experiment.

**Table 1.** Ingredient analysis of raw soybeans and soybean meal, as fed basis

Item	Raw soybeans	Soybean meal
Trypsin inhibitor units, TIU/mg	57.38	2.99
Dry matter, %	88.96	88.15
Ash, %	5.72	6.62
Crude protein, %	34.60	46.50
Crude fat, %	19.50	1.59
Acid detergent fiber, %	6.20	7.40
Neutral detergent fiber, %	7.50	7.10
Calcium, %	0.29	0.62
Phosphorus, %	0.52	0.73
*Indispensable amino acids, %*		
Arginine	2.49	3.25
Histidine	0.90	1.27
Isoleucine	1.70	2.24
Leucine	2.63	3.53
Lysine	2.29	2.99
Methionine	0.50	0.61
Phenylalanine	1.74	2.34
Threonine	1.32	1.72
Tryptophan	0.50	0.79
Valine	1.73	2.32
*Dispensable amino acids, %*		
Alanine	1.47	1.96
Aspartic acid	3.88	5.13
Cysteine	0.53	0.62
Glutamic acid	6.08	8.26
Glycine	1.48	1.90
Hydroxyproline	0.07	0.06
Proline	1.76	2.24
Serine	1.48	1.77
Taurine	0.13	0.13
Tyrosine	1.34	1.61

Raw soybeans and soybean meal were analyzed (**Table 1**) for moisture (method 930.15 of AOAC, 2007), ash (AOAC 942.05), crude protein (**CP**; AOAC 990.03), NDF (AOAC 2001.11), acid detergent fiber (**ADF**) based on Goering and Van Soest (1970), calcium and phosphorus (AOAC 985.01), crude fat (AOAC 2003.05), and crude fiber based on AOCS Ba 6a-05. The indispensable and indispensable amino acids composition of raw soybeans and soybean meal was determined by the Agricultural Experiment Station Chemical Laboratories at the University of Missouri–Columbia (Columbia, MO) by cation-exchange HPLC (L8900 Amino Acid Analyzer, Hitachi High-Technologies Corporation, Tokyo, Japan).

Trypsin inhibitor concentration in soybean meal and whole raw soybeans (Table 1), and complete feed (Table 2) was determined as previously described by Kim and Krishnan (2023). Briefly, in triplicate, 20 mg of dried and finely ground sample and 1 mL of 10 mM NaOH were added to a 2 mL centrifuge tube and then vortexed for 10 min. Samples were then subject to centrifugation at 16,000 × *g* for 10 min to isolate supernatant for measuring KTi activity. A reaction containing 200 µL of assay buffer (50 mM-Tris–HCL, pH 8.2 and 20 mM CaCl_2_), 500 µL of *N*α-Benzoyl-DL-arginine 4-nitroanilide hydrochloride (0.4 mg/mL), and 200 µL of trypsin (20 µg/mL) was performed for 10 min at 37 °C and then was terminated with the 200 µL of 30% acetic acid. The reaction was measured at an absorbance of 410 nm, and results were expressed as trypsin units inhibited per milligram of sample (TIU/mg).

**Table 2.** Diet composition, as fed basis

	Treatment, TIU/mg^8^				
Ingredients, %	0.99	2.23	3.07	6.49	9.38
Corn	64.79	64.89	64.35	63.31	75.87
Whole ground soybeans	-	1.63	4.86	11.12	20.25
Soybean meal	29.64	28.17	25.98	21.72	-
Soybean hulls	0.60	0.53	0.43	0.24	-
Soybean oil	2.85	2.63	2.22	1.42	-
Salt	0.40	0.40	0.40	0.40	0.40
Monocalcium phosphate 21%	0.64	0.65	0.65	0.66	1.04
Limestone	0.59	0.60	0.61	0.63	0.74
L-lysine HCl	0.10	0.10	0.10	0.10	0.59
DL-methionine	0.05	0.05	0.05	0.05	0.18
L-threonine	0.01	0.02	0.02	0.02	0.23
L-tryptophan	-	-	-	-	0.11
L-valine	-	-	-	-	0.15
L-isoleucine	-	-	-	-	0.11
Trace mineral premix^1^	0.15	0.15	0.15	0.15	0.15
Vitamin premix^2^	0.15	0.15	0.15	0.15	0.15
Phytase^3^	0.03	0.03	0.03	0.03	0.03
*Calculated composition*					
Dry matter, %	89.24	89.23	89.21	89.16	89.35
ME, kcal/kg	3435	3435	3435	3435	3435
g SID Lys:ME	2.85	2.85	2.85	2.85	2.85
Crude protein, %	18.53	18.42	18.47	18.56	15.45
Crude fat, %	5.59	5.67	5.83	6.14	6.59
ADF, %	3.91	3.88	3.88	3.88	3.07
NDF, %	7.41	7.40	7.40	7.40	7.41
Calcium, %	0.54	0.54	0.54	0.54	0.54
Phosphorus, %	0.52	0.52	0.52	0.52	0.52
SID Lysine, %	0.98	0.98	0.98	0.98	0.98
TIU/mg feed	0.89	1.77	3.55	6.99	11.55
*SID AA:Lys*					
Thr %	0.62	0.62	0.62	0.62	0.62
Met + Cys%	0.58	0.58	0.58	0.58	0.58
Trp %	0.24	0.24	0.23	0.23	0.23
Ile %	0.74	0.74	0.74	0.73	0.56
Val %	0.82	0.81	0.81	0.80	0.66
Leu %	1.54	1.53	1.52	1.51	1.13
Phe + Tyr%	1.42	1.41	1.40	1.40	0.93
*Analyzed composition*					
Trypsin inhibitor units/mg feed	0.99	2.23	3.07	6.49	9.38
Crude protein, %	17.7	17.7	16.2	17.1	13.6

^1^Trace mineral premix provided 22 mg Cu (as CuSO_4_), 220 mg Fe (as FeSO_4_), 0.4 mg I (as Ca(IO_3_)_2_), 52 mg Mn (as MnSO_4_), 220 mg Zn (as ZnSO_4_), and 0.4 mg Se (as Na_2_SeO_3_) per kilogram of diet.

^2^Vitamin premix provided the following per kilogram of diet: vitamin A (Vitamin A acetate), 2.2 mg; vitamin D3 (Cholecalciferol), 0.02 mg; vitamin E (d-α-Tocopheryl acetate), 37 mg; vitamin K (Menadione dimethylpyrimidinol bisulfite or Menadione nicotinamide bisulfite), 30 mg; vitamin B12 (Cyanocobalamin), 0.05 mg; riboflavin, 11 mg; niacin (Niacinamide or Nicotinic acid), 56 mg; and pantothenic acid (d-Calcium pantothenate), 27 mg.

^3^Optiphos PLUS 2500 G (Huvepharma, Peachtree City, GA) provided 250 phytase units/kg (0.125) available phosphorus.

ME, metabolizable energy; ADF, acid detergent fiber; NDF, neutral detergent fiber; SID, standardized ileal digestible; TIU, trypsin inhibitor units per unit of complete feed.

### Animals, housing, and experimental design

Forty-five gilts (PIC 1,050 × 337, PIC Genus, Hendersonville, TN) with a starting body weight (**BW**) of 39.7 ± 2.1 kg were randomly assigned to individual pens. Pens (1.41 m × 2.18 m) were half-slatted concrete floored, equipped with 2 mounted nipple waterers and contained one 2-head space covered feeder. Pigs were allowed *ad libitum* access to feed and water throughout the 27-d period. Pigs and feeders were weighed on days 0 and 21 of the trial to calculate average daily gain (**ADG**), average daily feed intake (**ADFI**), and feed efficiency (gain-to-feed, **G:F**).

On day 21 of the experiment, all gilts were placed into individual metabolism stalls (0.7 m × 1.5 m) equipped with one feeder and nipple waterer and contained slatted flooring. The metabolism stalls were housed in an environmentally controlled room and maintained at approximately 21 °C. Pigs were blocked by their day 0 initial BW and randomly assigned to metabolism stalls. Pigs were allowed to acclimate to the stalls for 72 h, and immediately after, total fecal and urine excretion were collected for 72 h. The experiment was completed on day 27 of the trial. Based on individual pig ADFI from day 1 to 21, pigs were offered ~95% of ad libitum access to feed, and daily feed allowance was split over 2 feedings at 0630 and 1800 hours. The individual pig feed amount that was allotted was adjusted prior to each feeding to maintain as close to ad libitum as possible while preventing wastage. Feed refusals were weighed back following acclimation and collection periods while in metabolism stalls. Pigs were allowed free access to water for the duration of the 6-d metabolism period. Pigs were weighed upon entry and exit to the metabolism stalls to calculate overall (days 0 to 27) ADG, ADFI, and G:F.

### Sample collection, preparation, and nitrogen balance

Representative feed samples of each dietary treatment were collected and stored at −20 °C until further analysis. Total feces and urine excretion were collected twice daily at 0630 and 1800 hours for 72 h and pooled by individual pig. Urine was captured in stainless steel buckets containing 15 mL of 6 N HCl to prevent nitrogen volatilization and microbial proliferation. Following each collection, total feces and urine were weighed and stored at −8 °C. At the conclusion of the experiment, urine was thawed, homogenized, subsampled, filtered with glass wool, and stored at 4 °C for subsequent analysis.

Total feces were thawed, weighed, and placed in a drying oven at 75 °C for 7 d, or until completely dried, and then weighed to determine dry matter (**DM**; method 930.15 of AOAC, 2007). Dried feces and feed samples were ground through a Wiley Mill with a 1-mm screen (Variable Speed Digital ED-5 Wiley Mill, Thomas Scientific, Swedesboro, NJ) and properly stored in a desiccator until further analytical analysis.

Feed, feces, and urine samples were analyzed in duplicates for nitrogen and CP (TruMac N, LECO Corporation, St. Joseph, MO; method 990.03 of AOAC, 2007) to calculate nitrogen balance. Filtered urine (1 g) was loaded into Leco crucibles lined with nickel liners. To avoid evaporation prior to combustion, urine was loaded into the carriers in small batches. Nitrogen values were deemed acceptable with a coefficient of variance of 1% or less. Nitrogen metabolism is expressed as both apparent total tract digestibility (**ATTD**) of nitrogen and grams of nitrogen intake per day (g/d). The following equations were utilized to calculate each, respectively:


$$\begin{array}{l} ATTD=\frac{\left[\left(N\;in\;feed\;\times Feed\;intake\right)-\left(N\;in\;feces\;\times Fecal\;output\right)\right]}{\left(N\;in\;feed\;\times Feed\;intake\right)}\times 100 \end{array}$$

$$\begin{array}{l} N\;intake,g/d=\;N\;in\;feed,g/d\times Feed\;intake,\;g/d\; \end{array}$$

Nitrogen balance was completed to determine excretion and retention of nitrogen. Nitrogen retention is expressed on a g/d, percent of intake, and percent of digestible nitrogen basis. The following equations were utilized:


$$\begin{array}{l} Total\;N\;excretion,g/d=Fecal\;N\;excretion,g/d+Urine\;N\;excretion,\;g/d \end{array}$$

$$\begin{array}{l} Total\;N\;retention,g/d=N\;intake,g/d-\;N\;excretion,\;g/d\; \end{array}$$

$$\begin{array}{l} N\;Retention\;\left(\%\;intake\right)=\frac{N\;retention,\;g/d}{N\;intake,\;g/d}\;\times \;100 \end{array}$$

$$\begin{array}{l} N\;Retention\;\left(\%\;digestible\right)=\frac{N\;retention,\;g/d}{N\;digested,\;g/d}\;\times 100 \end{array}$$

## Statistical Analysis

Data were analyzed using SAS (version 9.4, SAS Institute, Inc., Cary, NC). The UNIVARIATE procedure was utilized to verify the normality of all data. Growth, digestibility, and nitrogen balance data were analyzed with PROC MIXED, with the model including the fixed effect of dietary treatment (TIU/mg level). Orthogonal polynomial contrasts for linear and quadratic effects were conducted using the GLM procedure to account for unequal spacing between treatments, using analyzed diet TIU/mg levels. The pig was considered the experimental unit for all parameters. Data are presented as least-squares (LS) means and pooled standard error of the mean (SEM). Differences in LS means were determined using the probability of difference (pdiff) option, and Tukey-Kramer adjustment was implemented to account for multiple comparisons. Reported data were considered significant if *P *≤ 0.05 and a tendency if 0.05 ≤ *P *≤ 0.10. In addition, daily TIU intakes were calculated and plotted against ADG and G:F. Linear regressions (R^2^) were then performed to assess these relationships.

## Results

### Diets and ingredient analysis

Soybean meal and whole ground raw soybean proximate analysis and amino acid profiles used in this experiment are presented in Table 1. As expected, whole ground raw soybeans had the highest TIU concentration (57.4 TIU/mg), while conventionally solvent-extracted soybean meal had a concentration of 2.99 TIU/mg. Compared with the soybean meal ingredient, ground raw soybeans were lower in CP but had a comparable amino acid composition. Crude fat concentration was greater in the raw soybeans; ADF, calcium, and phosphorus were lower compared with the soybean meal samples used in this experiment.

Complete feed from all dietary treatments were analyzed for CP and TIU activity and reported in Table 2. CP concentration was similar across all treatments, except for the diet with the highest TIU activity, which was lower. Analyzed TIU activities were similar to the formulated values. The analyses of the 5 dietary treatments resulted in a concentration of 0.99, 2.23, 3.07, 6.49, and 9.38 TIU/mg.

### Growth performance

**Figure 1.**
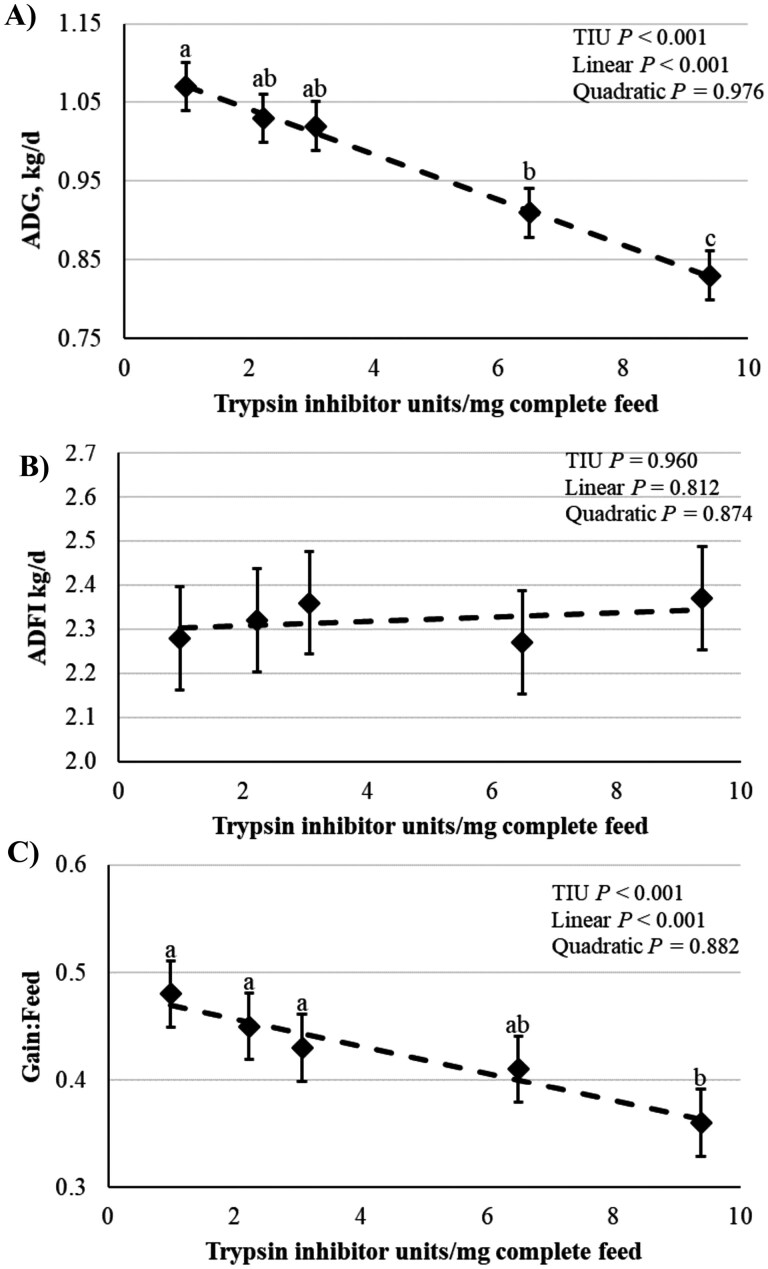
Effects of dietary soybean trypsin inhibitor concentrations (TIU/mg complete feed) on (**A**) ADG, (**B**) ADFI, and (**C**) Gain-to-Feed ratio over a 27-d test period. Different letters a,b,c represent *P* ≤ 0.05 between dietary treatments.

**Figure 2.**
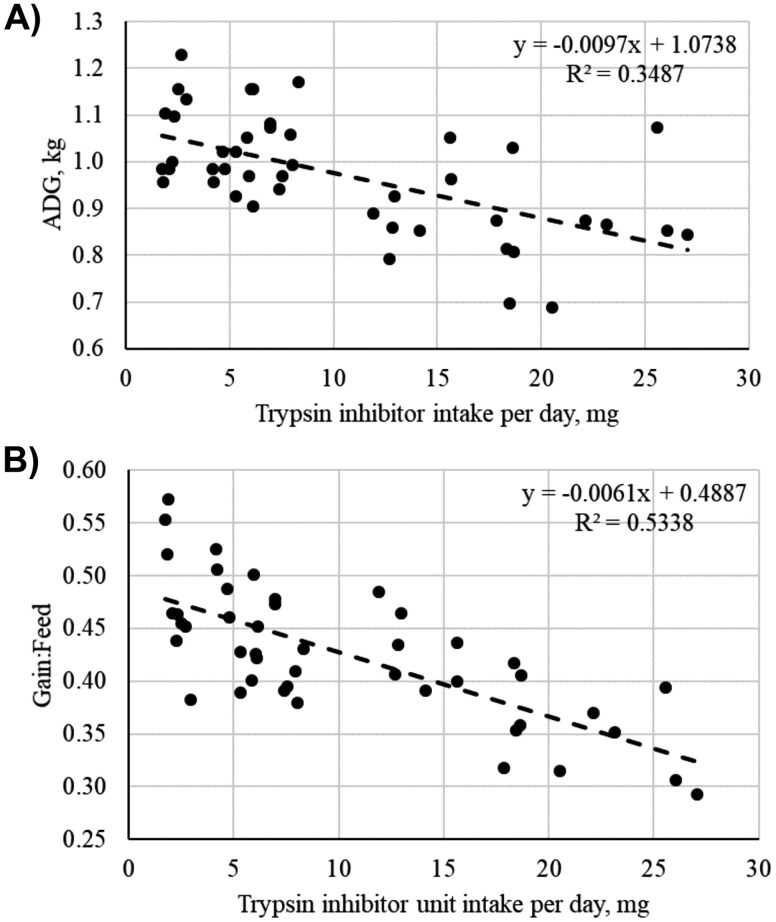
The correlation between daily soybean trypsin inhibitor (TIU) intake per day (mg TIU/d) and (**A**) Average daily gain (ADG) or (**B**) Gain-to-Feed ratio (G:F) in grower pigs fed over a 27-d experimental period.

As presented in Table 3, the initial BW of pigs were similar across treatments (*P *= 0.980). Pigs fed 9.38 TIU/mg had a 9.3% reduction in day 27 BW compared with pigs fed 0.99 TIU/mg (linear, *P *< 0.001). Final BW at day 27 of pigs fed 0.99, 2.23, or 3.07 TIU/mg were not different (*P *> 0.10). There were no differences (*P* > 0.10) in overall ADG between diets containing 0.99, 2.23, or 3.07 TIU/mg. However, ADG was reduced in pigs fed 6.49 and 9.38 TIU/mg at day 27 (15.0% and 22.4%, respectively) from the lowest 0.99 TIU/mg containing diet (linear; *P *< 0.001, Figure 1A). Dietary TIU concentration had no effect on ADFI over the 27-d period (*P *> 0.10, Figure 1B). Similar to ADG, as diet TIU increased, G:F linearly decreased (*P* < 0.001, Figure 1C). To further understand how TIU impacts pig performance, the relationship between daily TIU intake, ADG, and G:F was assessed over the 27-d experiment. As pig daily TIU intake increased, ADG was decreased (R^2^ = 0.349, Figure 2A). Likewise, G:F was also moderately negatively correlated with increasing daily TIU intake in grower pigs (R^2^ = 0.534, Figure 2B). Together, these data indicate that daily intakes of 10 mg TIU or more greatly compromise ADG and G:F in grower pigs.

**Table 3.** Body weights and nitrogen retention of pigs fed dietary treatments increasing in trypsin inhibitor units

	Treatment, TIU/mg^1^					SEM	*P* value		
Item^2^	0.99	2.23	3.07	6.49	9.38		Treatment	Linear	Quadratic
*Body weights*									
Start, kg	39.8	39.9	39.9	39.3	39.8	0.74	0.980	0.786	0.760
End, kg	68.7^a^	67.6^ab^	67.4^ab^	64.0^bc^	62.3^c^	0.93	<0.001	<0.001	0.818
*Nitrogen metabolism*									
ATTD Nitrogen, %	89.9^a^	89.3^a^	87.3^ab^	85.7^ab^	83.2^b^	1.24	0.003	<0.001	0.834
Nitrogen intake, g/d	61.3^a^	59.9^a^	58.8^a^	57.6^a^	45.9^b^	2.05	<0.001	<0.001	0.046
*Nitrogen excretion*									
Fecal, g/d	6.2	6.4	7.4	8.5	7.8	0.79	0.244	0.061	0.201
Urine, g/d	12.1^a^	12.2^a^	14.7^a^	14.1^a^	6.9^b^	1.23	<0.001	0.005	<0.001
Total, g/d	18.3^ab^	18.6^ab^	22.1^a^	22.6^a^	14.6^b^	1.32	<0.001	0.108	<0.001
*Nitrogen retention*									
g/d	43.0^a^	41.3^a^	36.7^ab^	35.0^ab^	31.3^b^	2.10	0.002	<0.001	0.493
% of intake	70.0^a^	68.9^ab^	62.2^ab^	60.5^b^	68.2^ab^	2.29	0.015	0.337	0.002
% of digested	77.^9ab^	77.1^ab^	71.2^b^	70.8^b^	81.9^a^	2.51	0.015	0.364	0.001

^1^TIU/mg = trypsin inhibitor unit per milligram of complete feed.

^2^ *n* = 9 pigs/treatment.

ATTD, apparent total tract digestibility.

^a,b,c^Means within row with differing superscripts indicate *P* ≤ 0.05.

### Nitrogen balance and digestibility

Digestibility and utilization of nitrogen are presented in Table 3. The ATTD of nitrogen was not different among treatments 0.99, 2.23, 3.07, and 6.49 TIU/mg (*P* > 0.10). However, pigs fed the diet with 9.38 TIU/mg had reduced ATTD of nitrogen coefficients compared with pigs fed diets with 0.99 or 2.23 TIU/mg (*P* < 0.001). Pigs fed the 9.38 TIU/mg diet had reduced nitrogen intake (g/d) compared with the other 4 treatment groups (*P* < 0.001), and a quadratic effect (*P* = 0.046) of nitrogen intake was observed.

Fecal nitrogen (g/d) excretion tended to increase (linear; *P* = 0.061) as the dietary TIU/mg level increased. Due to lower dietary CP content, excretion of nitrogen through urine (g/d) was reduced in the 9.38 TIU/mg diet, compared with the other 4 dietary treatments (*P* < 0.001). Taken together, pigs fed the diet containing 9.38 TIU/mg had reduced total nitrogen (g/d) excretion compared with diets with 3.07 or 6.49 TIU/mg (quadratic; *P* < 0.001), with diets containing 0.99 or 2.23 TIU/mg being intermediate.

Increasing TIU/mg in the diet linearly decreased nitrogen retention (g/d; *P* < 0.001). Pigs fed 9.38 TIU/mg retained 27.3% and 24.2% less nitrogen on a g/d basis compared with pigs fed the 0.99 or 2.23 TIU/mg diets, respectively (*P* < 0.001). As a percentage of nitrogen intake, pigs fed 6.49 TIU/mg diet had reduced nitrogen retention compared with pigs fed the 0.99 TIU/mg treatment (*P *< 0.05). On the basis of digestible nitrogen (%), a quadratic response was observed where pigs fed the highest amount of TIU/mg in the diet retained more nitrogen than pigs fed 3.07 or 6.49 TIU/mg, with 0.99 and 2.23 being intermediate (quadratic; *P *= 0.001).

## Discussion

Soybean-based protein ingredients are widely used in the swine industry to provide amino acids and protein to the diet of pigs in various stages of production. However, soybeans also contain naturally occurring antinutritional factors, including lectins, saponins, tannins, raffinose, stachyose, isoflavones, β-conglycinin, and 2 protease inhibitors known as KTi and BBI (Liener, 1994; Woyengo et al., 2017). The overall objective of the experiment presented was to determine the level at which TIU from soybeans in complete feed formulations would impact grower pig growth performance, digestibility, and nitrogen retention. Trypsin inhibitor proteins are naturally occurring and inevitably found in soybean products, as processing methods don’t always deactivate the inhibitor protein entirely (Herkelman et al., 1992). However, extended heat processing of soybean products may cause the formation of Maillard reaction products that limit amino acid bioavailability (Gonzalez-Vega et al., 2011).

It is widely acknowledged that high soybean TIU levels in complete diets inhibit the ability of pigs to digest and assimilate protein and amino acids (Liener, 1962; Yen et al., 1977). Amino acids are essential for synthesizing and maintaining proteins involved in enzymes, hormones, muscles, and other vital biological processes and tissues. Consequently, pigs fed soybean protein with high TIU levels may experience reduced BW gains and feed intakes. Early research in the area of trypsin inhibitor proteins and other antinutritional compounds in soybeans and production animal outcomes typically compared soybean variants with genetically known amounts of trypsin inhibitor concentrations on growth performance and nutrient digestibility of swine (Kakade et al., 1972; Yen et al., 1977; Cook et al., 1988; Herkelman et al., 1992; Zeamer et al., 2021) and other animal models like poultry (Palacios et al., 2004) and rats (Yen et al., 1971), where consistent reductions in daily weight gain and feed efficiency are observed.

To our knowledge, there is limited published research in pigs that has examined the titration of TIU in complete feed. Royer et al. (2022) reported in grower-finisher age pigs fed diets formulated from 0.7 to 4.8 TIU/mg with raw full-fat soybeans, that live weight linearly decreased with the ingestion of soybean-based trypsin inhibitor. Although the levels fed in this experiment were greater (up to 9.38 TIU/mg), our data agrees with Royer et al. (2022) because a linear decrease in ADG over the 27-d test period was observed.

In a recent experiment comparing conventional soybean meal to alternative soybean varieties bred to remove KTi, lectins, and P34 protein from the seed, fed to pigs from 8 to 25 kg BW, the authors reported no differences or reduced growth rates, ADFI, and feed efficiency (Zeamer et al., 2021). In a similar study, Cook et al. (1988) compared solvent-extracted soybean meal to soybeans bred for either low or high levels of KTi on both nursery and late grower pigs. These authors indicated reductions of ADG and G:F of 51.6% and 53.3%, respectively, in 7 kg nursery pigs, while a 12.6% reduction in ADG, and no change to G:F in 67 kg grower pigs, when fed a high KTi soybean variant compared with conventional soybean meal. In the present experiment, as expected, growth rates and feed efficiency decreased as TIU/mg levels increased from 0.99 to 9.38 TIU/mg complete feed. Compared with the control 0.99 TIU/mg treatment, overall ADG was decreased by 15% and 22.5% in the 6.49 and 9.38 TIU/mg treatments, respectively. Likewise, pigs fed the highest level of TIU (9.38 TIU/mg) had a 25% reduction in feed efficiency. These results corroborate Royer et al. (2022), where weight gain and feed efficiency were both reduced at and above 3.2 TIU/mg in growing pigs starting at approximately 30 kg body weight. Additionally, these reductions in ADG and G:F performance parameters were particularly evident in the grower pigs used within this experiment when daily TIU intakes exceeded 10 mg.

Consumption of diets containing high levels of TIU has been reported to have inconsistent effects on ADFI and may depend on the age of the pig and the TIU levels of the diet. Across multiple experiments, Royer et al. (2022) reported no differences in pigs fed 0.86 to 3.5 TIU/mg, an 8% reduction in ADFI when fed diets containing greater than 2.79 TIU/mg or 4.0 TIU/mg. Additionally, Cook et al. (1988) reported that feed intake was not different among treatments in both 7 kg pigs and 67 kg pigs when fed diets containing soybeans bred to have either high KTi or low KTi activity. In partial agreement with these authors, over the 27-d test period conducted in similar starting weight pigs, this experiment reported no differences in ADFI. The lack of feed intake response to dietary TIU level across studies may be due to differences in pig genetics, health status, or age. In nursery-age pigs, feed intake is drastically reduced as dietary TIU increased from 0.38 to 5.79 TIU/mg (Nisley et al., 2024). Crenshaw and Danielson (1985) investigated the interaction between the maturity age of pigs and raw soybeans in formulation and utilized pigs with a starting BW of 23, 45, and 68 kg and fed either soybean meal or raw soybeans. Those authors concluded that regardless of age and weight of pigs, feeding raw soybeans will reduce performance, indicating no interaction. This is likely due to the high concentrations of antinutritional compounds found in non-heat-treated soybeans (Jimenez et al., 1963; Herkelman et al., 1992). This is also in agreement with Young (1967), who replaced soybean meal with raw ground soybeans in ~5% increments between treatments, while holding constant CP content, in grower pigs across 2 trials. When pigs were fed 22.15% raw ground soybeans from 22 to ~85 kg, compared with pigs fed a diet containing only soybean meal, there were reductions of 38.4% in ADG, 38.9% in feed conversion ratio (FCR; Feed:Gain), while there was no change to ADFI.

High levels of TIU in the diet result in the inability of the pig to properly and efficiently digest protein (Liener, 1962). Antinutritional compounds, especially TIU, are inevitably found in soybeans and soybean meal; thus, digestibility may be affected (Liener, 1976). Using 24 kg grower pigs, Herkelman et al. (1992) compared heat treatment and soybean variety (either conventional or low TIU variant) to conventional soybean meal. Individual ingredients were analyzed for TIU/mg, whereby raw conventional soybeans contained 20.9 TIU/mg, raw low TIU soybeans contained 9.9 TIU/mg, and soybean meal contained 2.5 TIU/mg. These authors reported reductions of 44.5% for ADG and 44.8% for FCR when pigs were fed conventional, non-heat-treated soybeans to soybean meal. These authors credit the diet TIU content to the difference in performance, due to the decrease in the digestibility of amino acids. Consistently, all indispensable and dispensable amino acids resulted in reduced digestibility in raw conventional soybeans compared with soybean meal, and resulted in a 30.8% reduction in apparent ileal digestibility (AID) of nitrogen between the 2 treatment groups (Herkelman et al., 1992). More recently, Goebel and Stein (2011) utilized similar treatments and investigated AID and SID of amino acids using either conventional or low KTi soybeans, processed at low or high temperatures, and compared these to a soybean meal control. Feeding low-temperature processed conventional soybeans reduced AID of CP by 39.7% compared with soybean meal in 11.1 kg pigs. Although a different parameter of digestibility is presented herein, in alignment with these studies, we report a 7.5% linear decrease in ATTD of nitrogen between our lowest and highest treatment groups (0.99 and 9.38 TIU/mg). Likewise, Combs et al. (1967) investigated different heat treatments on soybeans and utilized soybean meal as a control. These authors used 8-wk-old pigs and reported digestibility of CP was reduced by 27.6% when pigs were fed nonheated soybeans compared with soybean meal. Similar trends of unprocessed soybeans, soybean varieties, and TIU concentrations have been reported in rats (Kakade et al., 1973), hamsters (Hasdai and Liener, 1983), and poultry (Clarke and Wiseman, 2005). The reduction in protein digestion is often accompanied by pancreatic hypertrophy, as animals overproduce protease digestive enzyme secretions (Schneeman et al., 1977). This is commonly reported in many species, such as poultry (Clarke and Wiseman, 2005), rats (Kakade et al., 1973; Schneeman et al., 1977; Grant et al., 1995), and hamsters (Hasdai and Liener, 1983); however, the pancreatic hypertrophy response is less clear in swine (Yen et al., 1977).

To further understand the impact of varying dietary TIU levels in growing pigs, total nitrogen balance was determined after pigs were on test diets for 21 d. Over a series of studies, Yen et al. (1974) investigated the effects of soybean variety on nitrogen excretion and retention in 10 to 20-kg pigs and found that pigs fed a diet containing soybean meal excreted 60.8% less fecal and 34% less urinary nitrogen than pigs fed raw soybeans, resulting in 49.2% more nitrogen retained. In rats, utilizing the same soybean varieties, Yen et al. (1971) reported a 19.2% reduction in retained nitrogen per day. The data presented in the current experiment are in alignment with these studies, where pigs fed diets higher in TIU/mg, achieved using raw soybeans, excreted more nitrogen, driven by urinary output up to treatment 6.49 TIU/mg. At the highest treatment of 9.38 TIU/mg, total nitrogen excretion was numerically the lowest; however, pigs on this dietary treatment seemed to retain a higher percentage of digested nitrogen compared with the other treatment groups. This quadratic effect is likely a consequence of the CP content of the diets in which consumption of nitrogen (g/d) was less in our pigs fed 9.38 TIU/mg than the other 4 treatment groups and where pigs fed this diet may have been retaining and utilizing amino acids for maintenance (Otto et al., 2003; Deng et al., 2007) resulting in a higher nitrogen retention coefficient. Otto et al. (2003) investigated the impact of CP content, with the inclusion of crystalline amino acids, on nitrogen retention in 45 kg pigs. In agreement with the data presented herein relative to nitrogen intake and absorption, pigs fed diets containing 6% CP retained 24.3% and 19.2% more nitrogen, respectively, compared with a diet containing 15% CP. Collectively, this indicates efficient utilization of consumed nitrogen and amino acids on a low CP diet. Moreover, we acknowledge that our highest TIU diet (9.38 TIU/mg) had lower CP digestibility and higher nitrogen retention (% of digested) due to being formulated at a low CP level.

With variation in soybean seed varieties and protein processing, diets utilizing soybean protein ingredients will contain varying amounts of antinutritional factors that can impede animal performance. Herein, we investigated the effects of various levels of soybean trypsin inhibitor in grower pig complete feed. It was hypothesized that dietary concentrations above 3 TIU/mg would attenuate pig growth performance and feed efficiency by reducing protein digestibility and retention. Pig growth rates, feed efficiency, and nitrogen digestibility and retention were negatively affected as dietary trypsin inhibitor intakes increased. The data presented in this experiment suggests that caution should be taken when diets are formulated above 3.0 TIU/mg if producers want to optimize pig performance. Ultimately, understanding the influence of trypsin inhibitor content and other antinutritional compounds innate to soybeans is imperative as producers continue to use soybean meal, and explore the possibility of incorporating whole soybeans into diet formulation for growing pigs.

## Abbreviations

ADF: acid detergent fiber
ADFI: average daily feed intake
ADG: average daily gain
AID: apparent ileal digestibility
AOAC: Association of Official Analytical Chemists
ATTD: apparent total tract digestibility
BBi: Bowman Birk inhibitor
BW: body weight
CP: crude protein
DM: dry matter
FCR: feed conversion ratio (feed:gain)
G:F: feed efficiency (gain:feed)
KTi: Kunitz trypsin inhibitor
LS: least square
ME: metabolizable energy
NDF: neutral detergent fiber
NRC: National Research Council
SID: standard ileal digestibility
TIU: trypsin inhibitor units
